# Experiences with IL-1 blockade in systemic juvenile idiopathic arthritis – data from the German AID-registry

**DOI:** 10.1186/s12969-021-00510-8

**Published:** 2021-03-22

**Authors:** Elke Lainka, Melanie Baehr, Bernadette Raszka, Johannes-Peter Haas, Boris Hügle, Nadine Fischer, Dirk Foell, Claas Hinze, Elisabeth Weissbarth-Riedel, Tilmann Kallinich, Gerd Horneff, Daniel Windschall, Eggert Lilienthal, Tim Niehues, Ulrich Neudorf, Rainer Berendes, Rolf-Michael Küster, Prasad Thomas Oommen, Christoph Rietschel, Thomas Lutz, Frank Weller-Heinemann, Klaus Tenbrock, Georg Leonhard Heubner, Jens Klotsche, Helmut Wittkowski

**Affiliations:** 1 Department of Pediatric Rheumatology, University Children’s Hospital Essen, Essen, Germany; 2 German Center for Pediatric and Adolescent Rheumatology, Garmisch-Partenkirchen, Germany; 3 Department of Pediatric Rheumatology and Immunology, University of Muenster, Muenster, Germany; 4 Pediatric Rheumatology, University Children’s Hospital Hamburg-Eppendorf, Hamburg, Germany; 5 Department of Pediatric Pneumology, Immunology and Intensive Medicine and Center for Chronically Sick Children, Charité University Medicine Berlin and German Rheumatism Research Centre Berlin, Berlin, Germany; 6 Department of Pediatrics, Asklepios Clinic, Centre for Pediatric Rheumatology, St. Augustin and Medical Faculty, University of Cologne, Cologne, Germany; 7 Department of Pediatric Rheumatology, St. Josef Hospital, Sendenhorst, Germany; 8 Department of Pediatrics, Ruhr-University Bochum, Bochum, Germany; 9 HELIOS Children’s Hospital, Pediatric Immunology and Rheumatology, Krefeld, Germany; 10 Department of Pediatric Rheumatology, St. Marien’s Children’s Hospital Landshut, Landshut, Germany; 11 Orthopedics centre Altona and Pediatric practice Rissen, Hamburg, Germany; 12 Department of Pediatric Oncology, Hematology and Clinical Immunology, Center for Child and Adolescent Health, Medical Faculty, Heinrich-Heine-University Duesseldorf, Duesseldorf, Germany; 13 Department of Pediatrics, Clementine Children’s Hospital Frankfurt, Frankfurt, Germany; 14 Center for Pediatric and Adolescent Medicine/Pediatric Rheumatology, University Hospital Heidelberg, Heidelberg, Germany; 15 Division of Pediatric Rheumatology, Prof. Hess Children’s Hospital, Bremen, Germany; 16 Department of Pediatric Pneumology, Allergology and Immunology, RWTH Aachen, Aachen, Germany; 17 Department of Pediatrics, Municipal Hospital Dresden, Dresden, Germany; 18 German Rheumatism Research Centre Berlin, Berlin, Germany

**Keywords:** Systemic juvenile idiopathic arthritis, Autoinflammatory disease, Proinflammatory cytokines, Interleukin-1, Anakinra, Canakinumab

## Abstract

**Background:**

Systemic juvenile idiopathic arthritis (sJIA) is a complex disease with dysregulation of the innate immune system driven by cytokines. A major role is ascribed to interleukin-1β (IL-1β), supporting the autoinflammatory character of the disease and offering an effective blocking mechanism for treatment. Here we present clinical practice data from the German AID-registry for patients treated with IL-1 inhibition (IL-1i).

**Methods:**

In 2009 a clinical and research consortium (AID-Net) was established, including an online AID-registry. Patients with documented sJIA diagnosis were identified. Data for this retrospective IL-1i study were recorded by 17 centers. Response to treatment was evaluated according to Wallace criteria and additionally by an own classifying clinical response system.

**Results:**

In 6 years, 202 patients with confirmed sJIA were recorded in the AID-registry. Out of these, 111 children received therapy with Anakinra (ANA) (*n* = 84, 39 f) and/or Canakinumab (CANA) (*n* = 27, 15 f) at a median age of 8.7 y (range 0.6–19.1). During the first 12 months 75/111 (ANA 55, CANA 20) patients were evaluated according to Wallace criteria (achievement of inactive disease 28/55 and 17/20, remission over 6 months under medication 13/55 and 7/20 cases). Over the whole period of time, clinical response was preserved in the majority of patients (ANA 54/80, CANA 20/27). Arthritis mostly persisted in polyarticular (PA) courses. During treatment with IL-1i concomitant medication could be tapered in about 15%. IL-1i was discontinued in 59/111 patients. 45 (15) adverse events (AE)s in ANA (CANA) treated patients (19.7 (26.6) AE/100 ANA (CANA) exposure years, 95%CI: 14.4–26.4 (14.9–43.9)) were reported.

**Conclusion:**

In a large cohort of sJIA patients from Germany, we can confirm an overall favorable clinical response to both available IL-1 blocking agents. IL-1i was well tolerated with acceptable safety and effectiveness in a real-life clinical setting.

## Background

The underlying etiology for systemic juvenile idiopathic arthritis (sJIA) is thought to be multigenic, and both environmental and genetic factors are implicated. The clinical picture is characterized by daily spiking fever, arthritis and rash, serositis, lymphadenopathy or hepatomegaly [1]. Presentation can be variable and arthritis can be a later feature [2]. Currently, sJIA is classified by the International League of Associations for Rheumatology (ILAR) as a category of juvenile idiopathic arthritis (JIA) and represents about 4,4% of 8096 JIA cases in the German National Pediatric Rheumatologic Database in 2017 [[3], unpublished data from German Rheumatism Research Centre (DRFZ)].

SJIA has been treated with corticosteroids (CO), nonsteroidal anti-inflammatory drugs (NSAIDs) and disease-modifying antirheumatic drugs (DMARDs) like methotrexate (MTX) [4, 5]. Cyclosporine A as calcineurin inhibitor is considered as an alternative cheaper drug, e.g. in resource-poor setting [6]. New strategies in diagnosing and managing sJIA as autoinflammatory disease (AID) have been recently published, and involve expensive cytokine-directed therapies against interleukin-1 (IL-1) and interleukin-6 (IL-6) [4, 7, 8]. Both treatment strategies are applied increasingly in sJIA cohorts since market approval of the available drugs. In 2013, the proportion of patients with sJIA who were treated with biologicals in Germany was reported to have increased to 20% [9]. Recently, sJIA patients of AID-registry, treated with tocilizumab (TCZ) as anti-IL-6 blockade, reached inactive disease or remission after 1 year of treatment in 75% of patients [10]. Available and approved IL-1 blocking agents are Anakinra (ANA) (IL1-receptor antagonist, for children older than 8 months and ≥ 10 kg weight, half-life 4–6 h) and Canakinumab (CANA) (anti-human-IL-1beta monoclonal antibody, for children older than 2 years, half-life 26 days) [11–13]. If the initial autoinflammation with dysregulation of innate immunity is not stopped, phenotype changes to a dysregulation of adaptive immunity with dominance of destructive arthritis. Early biological treatment has been proposed to foster a favorable long-term outcome in sJIA patients, leading to the hypothesis of a “window of opportunity” [14–16].

We present results of the AID-registry out of clinical practice on clinical characteristics, disease activity, clinical response, inflammatory parameters, and safety of children with sJIA in Germany receiving anti-IL-1 treatment in Germany.

## Methods

### Translational AID-net

The AID-registry is part of the AID-Net (Network for autoinflammatory diseases), a research initiative funded by the German Federal Ministry of Education and Research (BMBF) und supported by the German Rheumatism Research Centre (DRFZ) and the German society for pediatric rheumatology (GKJR). Retrospective patient data are documented following a pseudonymisation procedure in an online registry; additionally, patient material is collected and stored in a biomaterial bank for serum and plasma [17].

### Patients

From 2009 to 2017, 248 patients with new onset or already established diagnosis of sJIA, which was diagnosed by pediatric rheumatologists, were included in the AID-registry. Demographic information, clinical data, and blood samples (serum and EDTA blood) for biomarkers are collected at study enrollment and longitudinally documented during follow-up visits.

### Inclusion criteria (Fig. 1)

We included sJIA patients who fulfilled ILAR classification criteria [3], but also subjects meeting other definitions for sJIA, as confirmed by pediatric rheumatologists [4, 7, 8, 18, 19]. For study inclusion, a minimum number of 2 visits per year were necessary for each patient until the end of 2015.

**Fig. 1:**
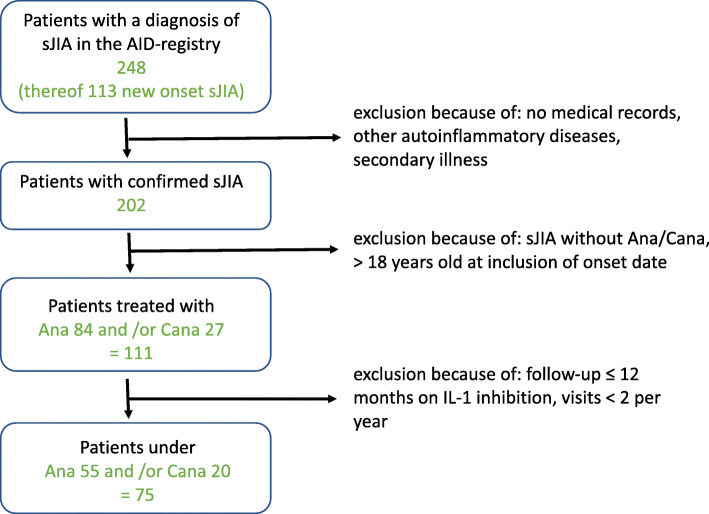
Flow chart for the inclusion criteria with a diagnosis of sJIA confirmed by pediatric rheumatology centers

Different clinical phenotypes of sJIA were defined: monocyclic (MC) means a persistent flare, after one episode the disease is inactive; polycyclic (PC) corresponds to recurrent flares (active disease alternating with inactive disease); polyarticular (PA) implies flares with arthritis in > 4 joints and a less pronounced systemic inflammation over time [20].

Concomitant medication before, during and after IL-1 inhibition (IL-1i), adverse events (AEs) and predictive factors for clinical response were reported.

Blood cell count, creatinine, aspartate aminotransferase, alanine aminotransferase and inflammation parameters like C-reactive protein (CRP, normal < 5 mg/l), erythrocyte sedimentation rate (ESR, mm after 1 h; normal male 3–15 mm, female 6–20 mm) and serum amyloid A (SAA, normal < 10 mg/l) were determined and reported in the treating centers. S100A12 (normal < 150 ng/ml) serum levels were measured in samples centrally stored at the AID-biobank, University of Muenster, as reported previously [21].

### Assessment of treatment response

Clinical response was reported at each visit in 3 categories: (1) a good response was considered if signs and symptoms of active disease (fever, rash, adenopathy, hepatosplenomegaly, serositis, arthritis) resolved, and if the inflammatory markers CRP and ESR improved by at least 50% and if this response was maintained for at least 6 months, (2) a transient response was considered if there was an initial good response for at least 2 months but then recurrence of inflammatory symptoms, and (3) a poor response was considered if clinical signs and symptoms did not resolve or inflammatory parameters were not reduced sufficiently. Non-response was determined over the whole observation time and described patients with clinical poor response who switched from IL-1 inhibition to another medication.

Response after 12 months was evaluated based on Wallace criteria, differentiating between active disease (AD), inactive disease (ID) and clinical remission on medication (CRM). Wallace et al. defined ID by the following criteria: no active arthritis, no fever, no exanthema, no serositis, no splenomegaly, no lymphadenopathy, no active uveitis, normal ESR and CRP, no disease activity in physician’s report. CRM was defined as ID for at least 6 months. Active disease (AD) described a visit with at least one symptom of the above and/or increased inflammatory parameters [22].

### Statistical analysis

Descriptive analyses included median with range, standard deviation for continuously distributed parameters as well as absolute and relative frequencies for categorical data. Anonymized data analysis was implemented in IBM SPSS Statistics 19 (SPSS Inc., Chicago, IL, USA). Inflammation parameters before and during treatment were compared by Wilcoxon sign rank test for matched pairs. The number of AEs was reported by 100 years under ANA or CANA treatment. The confidence interval (CI) for the rate per 100 treatment years was estimated by exact Poisson intervals.

### Ethics commission

The AID-registry has been approved by the ethics committees and the data protection responsibles at the University of Duisburg-Essen and Muenster, as well as the Medical Association Nordrhein in Duesseldorf. Parents, children between 9 and 13 years of age and young patients aged ≥14 years provide informed consent.

## Results

A total of 202/248 sJIA children in the AID-registry were enrolled in the study after exclusion of cases with secondary diagnoses or insufficient medical records. A total of 111/202 (55%) sJIA children were treated with ANA or CANA within the observation interval and were longitudinally documented in follow-up (Fig. 1). Altogether, for ANA/CANA 1595/706 visits (per patient median 15/23 (range 3–82) visits) and among these 565/263 visits (per patient median 5/5.5 (range 1–42) visits) during IL-1i were analyzed. Patients fulfilling these inclusion criteria were included from the following 17 AID-Net centers: Garmisch-Partenkirchen (*n* = 45), Muenster (*n* = 21), Berlin (*n* = 9), Hamburg (*n* = 9), St. Augustin (*n* = 7), Sendenhorst (*n* = 3), Bochum (*n* = 3), Essen, Hamburg-Altona, Krefeld, Landshut (each *n* = 2), Aachen, Bremen, Dresden, Duesseldorf, Frankfurt, Heidelberg (each *n* = 1). The decision to start or to stop treatment and the choice of biological agent was made by the participating center.

### Total population (*n* = 248)

**Fig. 2:**
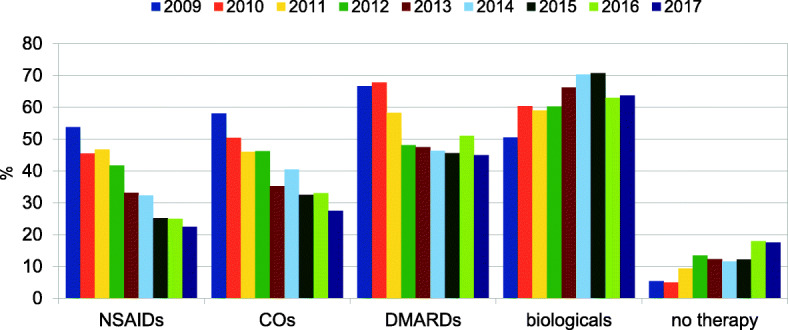
Annual examination of medication categories from all sJIA patients (independent of disease activity) in the AID-registry, *n* = 248

**Fig. 3:**
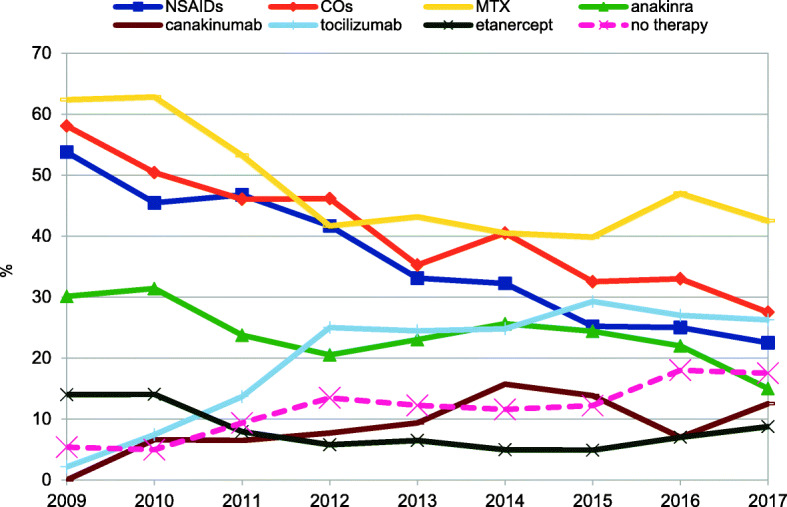
Annual examination of most received drugs from all sJIA patients in the AID-registry, *n* = 248

We performed an annual therapy evaluation of all children with new onset (*n* = 113) or already established (*n* = 135) diagnosis of sJIA. The following both diagrams show, which drugs were preferred in the treatment of sJIA any time (Figs. 2 and 3).

The choice of NSAIDs and glucocorticoids (CO) has been reduced by almost half in the last 9 years. The biological agents have been used most frequently since 2010. MTX continues to be used for more than 35% of all combinations with biologics. Biologics with DMARDs (+/− CO) was further preferred for sJIA with high disease activity. Over the entire period the category “no therapy” grew because of increase in remission.

### New onset sJIA (*n* = 113)

Every year 10–23 (4–9%) children were new diagnosed per year. 78% (88/113) new diagnosed children received NSAIDs, CO and/or DMARDs as the first line therapy. Second-line therapy included the use of 58% biological agents mostly in combination therapy. About 50% of children received biologicals in mono- or combination therapy as third-, fourth- or fifth-line therapy. Biologicals were initiated in the first year of treatment in 60% (68/113) of cases. In this group, 38% (26/68) biological agents (mostly in combination) were started as a first line therapy. 26 / 35 / 50 out of 68 children received biological agents as first line treatment / in the first month / in the first 3 months of therapy.

### Our patient cohort

One hundred eleven out of 202 (55%) enrolled children (57 m, 54 f) with sJIA were included, 84 patients treated with ANA (median age at diagnosis of 5.5 y; range 0.5–17.5) and 27 patients treated with CANA (7.4 y; range 1.8–17.3), respectively. Therapy with ANA and CANA was started at a median age of 6.8 y (range 0.6–19.1) and 8.7 y (range 2.2–19.1). Duration of IL-1i was longer in ANA vs. CANA treated patients: (34 mo; range 6–116) vs. (16 mo; range 4–58) (Table 1). First line treatment with anti-IL1i, without concomitant treatment, was only realized in one patient treated with CANA.

**Table 1:** Patient characteristics and disease courses

	ANA *n* = 84	CANA *n* = 27		ANA *n* = 80^a^	CANA *n* = 26^a^
**Age at diagnosis**	**5.5 years**	**7.4 years**			
**Symptoms before IL-1i**			**under IL-1i**		
Fever	90%	85%		28%	27%
Skin involvement	71%	81%		25%	33%
- Joint involvement* (arthritis/arthralgia)	59 70% / 19 23%	23 85% / 16 59%		37 46% / 21 26%	11 41% / 5 19%
- Abdominal	30%	70%		13%	26%
- Serositis	17%	11%		4%	4%
**Duration of initial therapy to IL-1i**	**15 months**	**15 months**			
**Age at start of IL-1i**	**6.8 years**	**8.7 years**			
**Duration of IL-1i**	**34 months**	**16 months**			
**Disease course**					
MC	7 (8%)	0 (0%)			
PC	36 (43%)	14 (52%)			
PA	41 (49%)	13 (48%)			
Joint involvement*	polyarthritis 41 (49%)	13 (48%)		25 (31%)	7 (27%)
	oligoarthritis 18 (21%)	8 (30%)		12 (15%)	4 (15%)
	arthralgia 19 (23%)	16 (59%)		21 (26%)	5 (19%)
	none 6 (7%)	0 (0%)		22 (28%)	10 (38%)

^a^symptoms unsatisfactory recorded

(IL-1i IL-1 inhibition, MC monocyclic, PC polycyclic, PA polyarticular)

*Specification of joint involvement

### Clinical and laboratory parameters

Systemic symptoms (ANA/CANA 84/27 children) before the start of IL-1i included mostly fever, arthritis, arthralgia, serositis, abdominal and skin involvement (Table 1). During IL-1i (ANA/CANA) a reduction or resolution of symptoms was achieved in 80/26 children. Arthritis (ANA/CANA 37/11, 46%/41%) persisted mostly in polyarticular (PA) courses (25 out of 41 / 7 out of 13, 68%/63%). No symptoms were observed in 23 patients (ANA/CANA 23%/19%). After initiation of IL-1i, inflammatory parameters declined irrespective of the disease course. In fact, 23/109 (21%) patients (ANA 16/83, CANA 7/26) receiving IL-1i showed normalized CRP-levels during therapy. Inflammation parameters were higher before and during therapy with ANA than with CANA (Table 2).

**Table 2:** Inflammation parameters before and during IL-1i, *p* value for test of change between inflammation parameters before and during treatment with ANA / CANA

	Before treatment		During treatment	***p***-value
Leukocytes /nl		Leukocytes /nl		
ANA *n* = 51	14.6 (3.5–49.0)	ANA *n* = 79	8.8. (2.4–21.8)	< 0.001
CANA *n* = 21	11.0 (5.4–28.3)	CANA *n* = 24	7.2 (4.9–15.4)	< 0.001
**CRP mg/l**		**CRP mg/l**		
ANA *n* = 51	48.7 (0–531)	ANA *n* = 78	17.1 (0–178.4)	< 0.001
CANA *n* = 20	16.3 (0–178.3)	CANA *n* = 26	6.4 (0–100)	0.014
**ESR (after 1 h) mm/h**		**ESR (after 1 h) mm/h**		
ANA *n* = 48	44.5 (1–106)	ANA *n* = 75	18 (1–125)	< 0.001
CANA *n* = 18	28.3 (7–100)	CANA *n* = 20	8.6 (1–101)	0.004
**SAA mg/l**		**SAA mg/l**		
ANA *n* = 9	357 (9.1–1510)	ANA *n* = 33	25.6 (0–1840)	0.014
CANA *n* = 6	26.4 (0.7–1840)	CANA *n* = 4	10.4 (0.5–1050)	0.663
**S100A12 ng/ml**		**S100A12 ng/ml**		
ANA *n* = 14	5740 (300–60,630)	ANA *n* = 51	220 (5–58,643)	0.021
CANA *n* = 14	2709 (0–60,630)	CANA *n* = 18	125 (0–5690)	0.001

### Response rate according to Wallace criteria

**Fig. 4:**
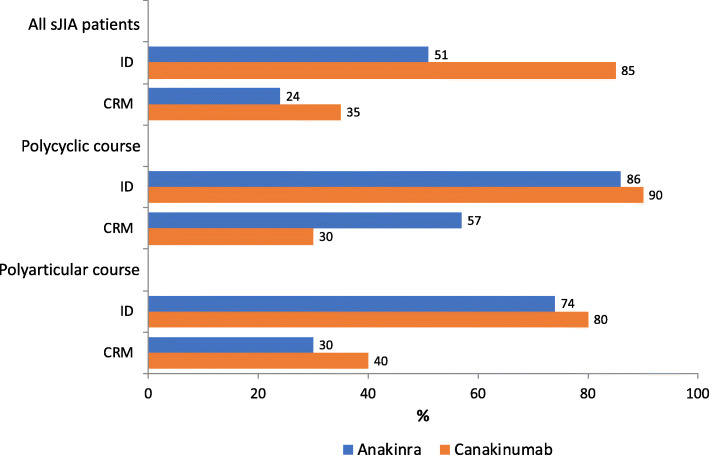
Proportion of patients in inactive disease (ID) and clinical remission on medication for at least 6 months (CRM) according to the Wallace criteria in all sJIA patients and patients on a polycyclic (PC) or polyarticular (PA) disease course (Monocyclic disease course (MC) was far too rare)

In 75/111 patients Wallace criteria could be applied. An ID could be determined for 28/55 (51%) children who received ANA and 17/20 (85%) who received CANA at any time within 12 months. Time period until first ID documentation was 89 d (range 7–260) for ANA and 56 d (range 2–217) for CANA. During IL-1i, 13/55 (24%) patients with ANA and 7/20 (35%) with CANA at any time achieved a state of remission on medication for at least 6 months. CRM and ID were more likely to be achieved in PC courses (Fig. 4).

### Clinical response (good/transient/poor)

A good clinical response to IL-1i according to our definition was shown in 68% of ANA (ANA 54/80) and 74% of CANA (CANA 20/27) patients. Poor clinical response was recorded for ANA in 27% (22/80) and for CANA in 11% (3/27) of cases during whole therapy *(*Table 3). Nine out of these poor response patients had PC, 2 had MC and 14 had PA disease courses. Indeed, PA course emerged as a negative predictor for clinical response (IL-1i, *p* = 0.018). Upon IL-1i PA courses more often showed persistence of arthritis and poor clinical response. Non-response was recorded for ANA in 19% (16/84) and for CANA in 4% (1/27) of cases so that treatment was changed.

**Table 3:** Clinical response rates for the entire follow-up time and at the 12-months follow-up after treatment initiation

sJIA	Good response		Transient response		Poor response	
	ANA	CANA	ANA	CANA	ANA	CANA
**Twelve months**	**79%** (50/63)	**95%** (21/22)			21% (13/63)	5% (1/22)
**Last follow-up**	**68%** (54/80)	**74%** (20/27)	5% (4/80)	15% (4/27)	27% (22/80)	11% (3/27)

### Concomitant therapy (Table 4)

**Fig. 5:**
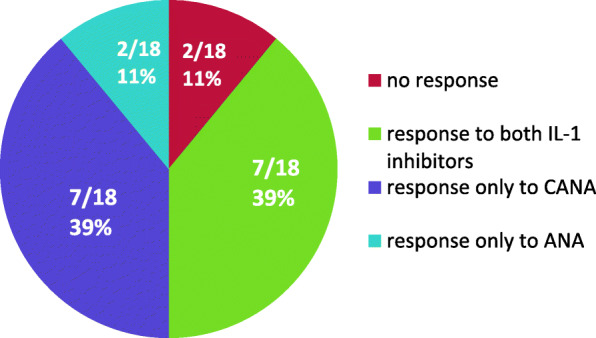
Clinical response to our definition in cases of switching IL-Ii from ANA to CANA (*n* = 18)

Before starting IL-1 blockade, 74% of patients were treated with DMARDs, 57% with non-IL-1 inhibiting biologicals, 78% with NSAIDs and 86% with CO. During IL-1i every other biological therapy was suspended, except in one patient, who was treated concomitantly with ETA. The rate of DMARDs therapy could be decreased to 58%, NSAIDs to 64%, and CO to 70%. As monotherapy ANA and CANA were used in 2/84 (2%) and 7/27 (26%) children. After discontinuation of IL-1i, no pharmaceutical treatment was applied in 6/51 (12%) and 1/8 (12.5%) patients reaching clinical remission off medication (CROM) according to Wallace criteria. Over the whole observation time, a switch from ANA to CANA (*n* = 18) was well tolerated (Fig. 5). Ten out of 18 patients the switch to CANA was initiated because of participation in a clinical study of Novartis (CACZ885G2402, EudraCT 2012–003054-92).

**Table 4:**
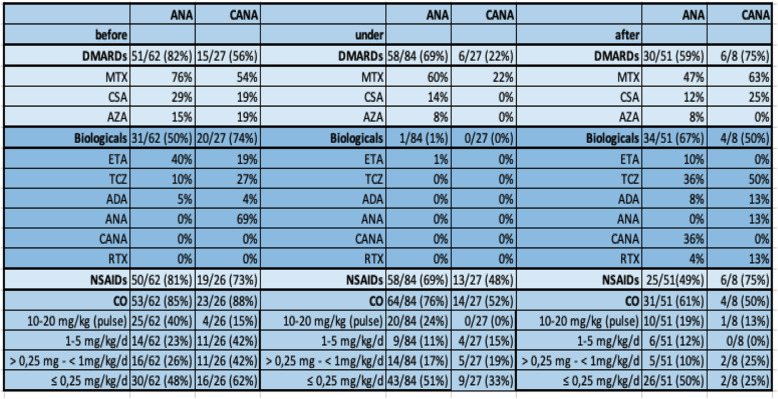
Concurrent medication: Children received disease-modifying antirheumatic drugs (DMARDs), [methotrexate (MTX), cyclosporine A (CSA), azathioprine (AZA)], biologicals [etanercept (ETA), tocilizumab (TCZ), adalimumab (ADA), rituximab (RTX)], nonsteroidal anti-inflammatory drugs (NSAIDs), and corticosteroids (CO).

### Discontinuation of IL-1i

59/111 (53%) patients discontinued IL-1i (ANA 51/84 61%, CANA 8/27 30%). Reasons for discontinuation of ANA were non-response (16/51), good clinical response (15/51), switch to CANA pediatric study of Novartis (CACZ885G2402, EudraCT 2012–003054-92) (10/51), short term on demand therapy (3/51), AEs (2/51), trypanophobia (2/51) or unknown reason (3/51). Reasons for discontinuation of CANA were acute disease exacerbation (4/8), good response (2/8), chronic impairment (1/8) or non-response (1/8).

### Drug tolerance

AEs were analyzed for 106 patients contributing 228 exposure years for ANA and 56.4 for CANA. Forty-five AEs (19.7 AE/100 ANA exposure years, 95%CI: 14.4–26.4) were reported in 43/78 (55%) patients including infections, elevated transaminases (at least two-fold increased), neutropenia, headache, trypanophobia and local site reactions *(*Table 5*)*. Fifteen adverse events (26.6 AE/100 CANA exposure years, 95%CI: 14.9–43.9) were reported in 15/26 (58%) patients including infections, elevated transaminases and headache. No macrophage activation syndrome (MAS), no amyloidosis, and no case of death were reported. In 4/84 (5%) AEs were the reason for stopping ANA administration: WHO toxicity II and III *n* = 2, trypanophobia *n* = 2. CANA therapy was not terminated because of AEs.

**Table 5:** Adverse event rates during treatment with ANA and CANA

	CANA	ANA
	sJIA	sJIA
	(***n*** = 26 patients, PY = 56.4)	(***n*** = 80 patients, PY = 228)
	Events; Rate per 100 PY (95% CI)	Events; Rate per 100 PY (95% CI)
**AE total**	**15; 26.6 (14.9; 43.9)**	**45; 19.7 (14.4; 26.4)**
Infections	6; 10.6 (3.9; 23.2)	15; 6.6 (3.7; 10.9)
Headache	5; 8.9 (2.9; 20.7)	8; 3.5 (1.5; 6.9)
Neutropenia	3; 5.3 (1.1; 15.5)	9; 3.9 (1.8; 7.5)
Increase of transaminases	6; 10.6 (3.9; 23.2)	11; 4.8 (2.4; 8.6)
Urticaria		2; 0.9 (0.1; 3.2)
Hematuria		4; 1.8 (0.5; 4.5)
Skin reaction		2; 0.9 (0.1; 3.2)
Proteinuria	2; 3.5 (0.4; 12.8)	4; 1.8 (0.5; 4.5)
Dizziness		
Local site reaction/ aversion	1; 1.8 (0.0; 9.9)	5; 2.2 (0.7; 5.1)
Nausea		1; 0.4 (0.0; 2.4)
Leucopenia		1; 0.4 (0.0; 2.4)
Thrombocytopenia	1; 1.8 (0.0; 9.9)	
Stomach pain	1; 1.8 (0.0; 9.9)	
Breast pain	1; 1.8 (0.0; 9.9)	
Nocturnal sweating		1; 0.4 (0.0; 2.4)
Acne		3; 1.3 (0.3; 3.8)

*PY* Person Years

## Discussion

SJIA represents a significant challenge for diagnosis and therapeutic strategies. Since approval of cytokine-directed therapies against IL-1 and IL-6, practical approaches of pediatric rheumatologists have changed, but nevertheless inherent questions concerning the best strategy – which blockade? Do we still need CO? Who will respond? Is there a window of opportunity? – are still pending. How can we harmonize treatment? Long-term follow-up in the AID-registry enables the report of results on choice of treatment, clinical response rates and safety of IL-1i in a real-world large independently funded cohort of well-characterized patients.

Prescription of biologicals in German comparator cohorts was reported by Horneff et al. for the BIKER registry (245 sJIA-patients) with 16% of patients treated with ANA and 9% with CANA in a time span from 2000 to 2015 [23]. The National Pediatric Rheumatologic Database from Germany, provides representative sociodemographic data and clinical characteristics, and recorded a proportion of 11% ANA treated and of 3% CANA treated patients from a total of 162 sJIA patients between 2011 and 2013 [9]. Even though direct comparison of these cohorts is not possible, after validation of sJIA diagnosis we report on 111 out of 202 sJIA-patients (55%) treated with IL-1i in our cohort between 2009 and 2015. This higher rate of biological treatment can mainly be ascribed to a difference in observation periods, including the years after approval of CANA for sJIA. While the overall rate of biological therapy in the AID-registry for sJIA-patients is high, their use in the first 3 months of treatment (22%) is still substantially lower [4].

Limitations of our analysis are varying quality of data documentation in different centers and by interobserver variability, so that we could not extract more formal response criteria from the registry, such as the modified pACR response criteria. Questionnaires like visual analog scales from patient or parents are missing. Disease activity, disease course and period of time until start of IL-1i varies significantly. Dosages of biological agents were not recorded, the complex role of co-medication could not be analyzed in detail, and the variable duration of follow-up may have had an impact on the recorded treatment outcomes. Furthermore 10 children discontinued ANA treatment because of recruitment for a clinical CANA trial.

Overall response to anti-IL1i in our cohort was good in 84% of patients within 1 year and thus comparable to previous studies [11, 12, 24, 25]. Additionally, a proportion of 60% reached ID and 27% CRM within 1 year, again comparable to other cohorts [26, 27]. Patients in AID-registry had longstanding and refractory disease. Rates of ID in comparable analyses ranged from 24 to 72% at long term follow up (2–3 years) [9, 23, 28]. To compare our results with IL-1i as first line treatment or with prospective treat-to-target approaches is not correct [29, 30]. First line treatment with anti-IL1i, without concomitant treatment, was only realized in one patient of our cohort.

Current treatment treat-to-target strategies for sJIA in Germany were published in 2018 by a PRO-KIND (projects for the classification, monitoring and therapy in pediatric rheumatology) group [4]. One aim should be to avoid or reduce CO. In the second trial for approval of CANA all patients received CANA to taper CO. Only in one third of the patients, CO could be discontinued, about half of the patients tolerated dose reduction [11, 12]. Initial concomitant treatment with CO was significantly less frequent with 50% in the TCZ treated AID-registry group and 44% in the TCZ treated or 45% in the IL-1i treated BIKER-registry group compared to our present study [10, 23] (Table 4). The differences may be explained by a longer time span between age of diagnosis and start of treatment and by the different use of medication as first line, second line or later treatment. Before starting IL-1i, MTX in BIKER-registry was used in 38% of the IL-1i cohort [23]. Meanwhile, MTX in our present study was used in 76% of the ANA and 54% of the CANA cohort (Table 4). During treatment CO, NSAIDs and DMARDs were reduced approximately by 10% with ANA and by 25–35% with CANA.

In a 5-year long term extension CANA study, it was shown that of the 128/177 (72.3%) patients on CO, 20 (15.6%) discontinued and 28 (22%) tapered. Overall, 75/177 (42%) children continuously took CANA and 58% discontinued mainly for inefficacy. 4% of patients discontinued CANA due to clinical remission [29]. In our AID-registry, 53% of patients finished IL-1i. After withdrawing IL-1i because of clinical response, 12% of patients received no further treatment. Vastert et al. started ANA in new-onset sJIA who were CO-naïve. At 3 months, 75% of patients achieved response while receiving ANA monotherapy and treatment could be stopped within 1 year, due to remission. However, in about one third of patients, concomitant therapy was required for maintenance of clinical response [14]. Ter Haar et al. recorded in the 5 years follow-up that ANA as first-line monotherapy resulted in 96% of patients with ID while 75% received no medication and 33% needed CO to achieve ID [30].

Infections, elevated transaminases, neutropenia, headache, local site reactions and trypanophobia were described in our cohort. Minimal AEs are frequently reported in roughly half of the patients but severe complications like MAS were not reported at all. Sota et al. described that the overall estimated rate of AE and SAEs was 8.4 per 100 patients per year for IL-1i in children and adults. 15% were classified as SAEs. The risk for AE tends to decrease over time from the start of IL-1i. No differences were detected between monotherapy and combination therapy [31]. In France, a high rate of AEs was described in 58% of adults and children. Injection-site reactions and liver toxicity were significantly more frequent in children than in adults. CANA showed better cutaneous tolerance than ANA but similar rates of non-cutaneous events [32]. Discontinuation of IL-1i because of AE was very rare [33]. Long term use of CANA described SAEs like sJIA flares, MAS and serious infections [29]. In the ANA-first line cohort, 4 (9.5%) patients developed MAS and 1 child died with pulmonary hypertension and central nervous system involvement [30]. Further studies showed, that MAS occurs even in patients whose sJIA is well controlled by treatment. Infections were the most common trigger factor [34].

Patients with sJIA are characterized by a marked and persistent activation of the innate immune system, but there is potential heterogeneity as suggested by the exquisite sensitivity to IL-1 blockade in a subset of patients as well as by differences in clinical course [7]. Patients with a PA course tend to lose systemic inflammatory activity switching to a more autoimmune phenotype [35]. The worst outcome among our present IL-1i and in our TCZ cohort was found for PA disease courses [10]. While the age at onset of disease, short disease duration, elevated neutrophil count, early response rate and ferritin level have been proposed to predict the response to IL-1i [30, 36, 37], we can confirm in the present study that PA disease course is a negative predictor of response to IL-1i. IL-1i may be considered mainly in patients with high systemic disease activity and lower active joint count [24, 36, 38]. In case of predominant PA appearance and/or lack of response to IL-1, initiation of or an early switch to IL-6 inhibition may be beneficial. Further treatment strategies featuring TNF-alpha- or JAK/STAT inhibition may be tested in the future. Positive predictors of a shorter time to remission have been published with oligoarticular onset, absence of active arthritis, ESR < 26 mm/h and no requirement for CO therapy [39].

The CARRA protocols will be evaluated during the FiRst-line Options for Systemic juvenile idiopathic arthritis Treatment (FROST) study, comparing CO, MTX, IL-1i, and IL-6 blockade [40]. This is an appropriate way to compare biological and non-biological management strategies [4, 14, 15]. Similar to CARRA protocols the PRO-KIND committee has defined consensus-based strategies to harmonize sJIA-treatment approaches in Germany with standardized treat-to target protocols [4]. There is growing evidence that IL-1i as first line treatment could facilitate lower doses and a shorter duration of CO therapy, higher effectiveness, and influence molecular disease patterns [37]. Whether early effective treatment of sJIA with biological agents affects its long-term disease course and reduces the risk of chronic articular course, is still discussed. Future studies should try to identify biomarkers of subclinical disease activity in order to optimize strategies for tapering and discontinuing therapy. Additional biomarkers are needed to risk stratify patients in order to start alternative therapies at an early stage [30].

## Conclusion

Out of 202 sJIA children reported in the German AID-registry, 111 (55%) were treated with ANA/CANA showing ID in 51%/85% and CRM in 24%/35% according to Wallace criteria after 1 year. During treatment with IL-1i, co-medication like CO, NSAIDs and DMARDs was reduced or discontinued. IL-1i was well tolerated with acceptable drug tolerance and effectiveness in a real-life clinical setting. Mild AEs were frequent. Further long-term studies especially comparing different treatment strategies are mandatory.

## Data Availability

The datasets analyzed during the current study available from the corresponding author on reasonable request.

## Abbreviations

AD: Active disease
ADA: Adalimumab
AE: Adverse event
AID: Autoinflammatory diseases
AID-NET: Network for autoinflammatory diseases
ANA: Anakinra
AZA: Azathioprine
BIKER: Biologicals in the pediatric rheumatology
BMBF: German Federal Ministry of Education and Research
CANA: Canakinumab
CARRA: Childhood Arthritis and Rheumatology Research Alliance
CI: Confidence interval
CO: Corticosteroids
CRM: Clinical remission under medication
CROM: Clinical remission off medication
CRP: C-reactive protein
CSA: Cyclosporine A
DMARD: Disease-modifying antirheumatic drug
DRFZ: German Rheumatism Research Centre
ESR: Erythrocyte sedimentation rate
ETA: Etanercept
GKJR: German Society for Pediatric Rheumatology
ID: Inactive disease
ILAR: International League of Associations for Rheumatology
IL-1, IL-6: Interleukin-1, − 6
IL-1ß: IL-1beta
IL-1i: IL-1 inhibition
JIA: Juvenile idiopathic arthritis
MAS: Macrophage activation syndrome
MC: Monocyclic
MTX: Methotrexate
NSAID: Nonsteroidal anti-inflammatory drug
PA: Polyarticular
PC: Polycyclic
PRO-KIND: Projects for classification, monitoring and therapy in paediatric rheumatology
PY: Person years
RTX: Rituximab
SAA: Serum amyloid A
sJIA: Systemic Juvenile Idiopathic Arthritis
TCZ: Tocilizumab
TNF: Tumor necrosis factor
s.c.: Subcutaneously
i.v.: Intravenously
p.o.: Per os
